# Trabecular bone density in middle-aged women with reproductive disorders

**DOI:** 10.1530/EC-23-0166

**Published:** 2023-09-29

**Authors:** Charissa van Zwol-Janssens, Aglaia Hage, Kim van der Ham, Birgitta K Velthuis, Ricardo P J Budde, Maria P H Koster, Arie Franx, Bart C J M Fauser, Eric Boersma, Daniel Bos, Joop S E Laven, Yvonne V Louwers, the CREW consortium

**Affiliations:** 1 Division of Reproductive Endocrinology and Infertility, Department of Obstetrics and Gynaecology, Erasmus University Medical Center, Rotterdam, the Netherlands; 2 Department of Radiology, University Medical Center Utrecht, University of Utrecht, Utrecht, the Netherlands; 3 Department of Radiology and Nuclear Medicine, Erasmus University Medical Center, Rotterdam, the Netherlands; 4 Department of Obstetrics and Gynaecology, Erasmus University Medical Center, Rotterdam, the Netherlands; 5 Department of Reproductive Medicine and Gynaecology, University Medical Center Utrecht & University of Utrecht, Utrecht, the Netherlands; 6 Department of Cardiology, Erasmus University Medical Center, Rotterdam, the Netherlands; 7 Department of Epidemiology, Erasmus University Medical Center, Rotterdam, the Netherlands

**Keywords:** bone density, premature ovarian insufficiency, preeclampsia, polycystic ovary syndrome

## Abstract

Besides age, estrogen exposure plays a crucial role in changes in bone density (BD) in women. Premature ovarian insufficiency (POI) and polycystic ovary syndrome (PCOS) are conditions in reproductive-aged women in which the exposure to estrogen is substantially different. Women with a history of preeclampsia (PE) are expected to have normal estrogen exposure. Within the CREw-IMAGO study, we investigated if trabecular BD is different in these women because of differences in the duration of estrogen exposure. Trabecular BD was measured in thoracic vertebrae on coronary CT scans. Women with a reduced estrogen exposure (POI) have a lower BD compared to women with an intermediate exposure (PE) (mean difference (MD) −26.8, 95% CI −37.2 to −16.3). Women with a prolonged estrogen exposure (PCOS) have the highest BD (MD 15.0, 95% CI 4.3–25.7). These results support the hypothesis that the duration of estrogen exposure in these women is associated with trabecular BD.

**Significance statement:**

Our results suggest that middle-aged women with PCOS have a higher BD and women with POI have a lower BD. We hypothesized that this is due to either a prolonged estrogen exposure, as seen in women with PCOS, or a reduced estrogen exposure, as in women with POI. In the counseling of women with reproductive disorders on long-term health issues, coronary CT provides a unique opportunity to assess both coronary artery calcium score for cardiovascular screening as well as trabecular BD.

## Introduction

Reproductive disorders, such as premature ovarian insufficiency (POI) and polycystic ovary syndrome (PCOS), are associated with hormonal and metabolic changes, which can impact changes in bone density (BD). Women with POI are at risk for failure to attain peak bone mass or for early loss of BD, due to estrogen deficiency at an early age (1, 2, 3, 4, 5). Consequently, women with POI are advised to use hormone replacement therapy (HRT) (6). In contrast to POI, the relation between PCOS and BD is less well-known. PCOS is associated with central obesity, dyslipidemia, hypertension, and insulin resistance (7), which all have been described to decrease BD (8, 9, 10, 11). However, hyperandrogenism, one of the key diagnostic criteria of PCOS, could have a protective effect on BD (12). Furthermore, PCOS is associated with prolonged menopause which could lead to longer estrogen exposure and may result in a delay of loss of BD (13, 14). Studies addressing BD in women with PCOS have shown conflicting results; some studies showed a decreased BD during the reproductive lifespan (15, 16), whilst a 21-year follow-up study of women aged 61–78 years revealed normal BD (17). Women with a history of preeclampsia (PE), who have estrogen exposure that is comparable to the general population, can be seen as an intermediate group in both estrogen exposure and BD. Studies have shown that women with a history of PE have a normal BD (18, 19, 20, 21).

A unique cohort of middle-aged women with POI, PCOS, and a history of PE underwent a coronary computed tomography (CCT) for cardiovascular screening. In this cohort, we aimed to investigate the association between these reproductive disorders, as a proxy for differences in the duration of estrogen exposure, and BD on CCTs.

## Materials and methods

Women previously diagnosed with either POI, PCOS, or PE underwent a CCT scan as part of the Cardiovascular Risk Profile: Imaging and Gender-Specific Disorders (CREw-IMAGO) study. The rationale and design of this study have been described in detail elsewhere (22). In short, this multicenter, cross-sectional study aimed to assess cardiovascular abnormalities by CCT in women above the age of 40 with a history of reproductive disorders (22). This study was approved by the ethics review board of the University Medical Center Utrecht (https://trialsearch.who.int/, unique identifier: NTR5531). Although the CCT was performed as part of cardiovascular risk profile, it also provides data on BD. In this secondary analysis, we assessed trabecular bone density (TBD) in women aged >40 years.

### Assessment of CT-based bone density

Our primary study outcome was TBD measured in Hounsfield units (HUs) on the non-contrast CCT scan, an opportunistic measurement that has been previously described by Budoff *et al.* (23). In short, we measured the BD in the center of the trabecular bone of three thoracic vertebrae (ranged T7–10) and calculated the mean. Scans were excluded if no adequate measurements could be performed, due to abnormalities in the vertebra, if no vertebra was depicted in the field of view, or if the non-contrast CT scan was missing. Three independent physicians (AH, CZ, and KH) measured the BD, and the intra- and interobserver analysis in 20 scans showed an excellent intraclass correlation of 0.979–0.991 and 0.967, respectively.

### Assessment covariates

Information on ethnicity, smoking, medical history, the use of oral contraceptive or HRT, and menopausal status was obtained from questionnaires. Women’s height, weight, waist circumference (WC), and blood pressure (BP) were measured. Central obesity was defined as WC ≥88 cm and hypertension as systolic BP >130 mmHg and diastolic BP >85 mmHg. Dyslipidemia was defined as LDL ≥3.37 mmol/L, triglycerides ≥1.7 mmol/L, or the use of statins. Insulin resistance was defined as HOMA-IR (glucose × insulin/22.5) above 2 or the use of insulin sensitizers.

### Statistical analysis

Demographic and clinical characteristics of women with a history of PE were compared by univariable analysis to women with POI or PCOS. An ANCOVA was used to observe a potential trend of TBD due to the duration of estrogen exposure corrected for age and BMI. POI was seen as a proxy for reduced estrogen exposure, PCOS for prolonged estrogen exposure, and PE was considered to be the intermediate group. Finally, linear regression models were used for sub-analysis to assess the impact of HRT, central obesity, dyslipidemia, hypertension, insulin resistance, and type 2 diabetes on TBD in the different groups. All statistical analyses were performed using IBM Statistical Package for the Social Sciences Statistics for Windows, version 27.

## Results

A total of 491 women were included in the CREw-IMAGO study. After exclusion (44 due to inadequate CT images), 93 women with POI, 262 women with a history of PE, and 92 women with PCOS were included in this study. The baseline characteristics of each group are presented in Table 1. The POI group was older than the PE group (median 48.2 years (interquartile range (IQR) 46.4–51.6) vs 46.1 years (IQR 43.5–49.2); *P* < 0.001). As expected, in the POI group, more women had ever used hormone replacement therapy compared to the PE group (72.1% vs 11.8%; *P* < 0.001).

**Table 1:** Patient characteristics of women with a history of reproductive disorders.

	POI (*n* = 93)	PE (*n* = 262)	PCOS (*n* = 92)	*P*-value^a^	*P*-value^b^
Age, years (IQR)	48.2 (46.4–51.6)	46.1 (43.5–49.2)	48.7 (46.7–53.6)	<0.001	<0.001
BMI, kg/m^2^ (s.d.)	25.3 (4.4)	27.5 (5.9)	28.4 (6.3)	<0.001	0.13
Waist circumference, cm (s.d.)	90.1 (11.4)	89.2 (13.2)	95.6 (16.1)	0.29	<0.001
Central obesity, *n* (%)	38 (55.9)	121 (49.8)	58 (73.4)	0.38	<0.001
Ethnicity NE, *n* (%)	90 (96.8)	244 (97.2)	79 (87.8)	0.42	<0.01
Hypertension, *n* (%)	54 (58.1)	145 (55.3)	53 (57.6)	0.65	0.71
Dyslipidemia, *n* (%)	46 (49.5)	95 (40.9)	49 (53.8)	0.16	0.04
Insulin resistance, *n* (%)	9 (56.3)	4 (3.2)	51 (70.8)	<0.001	<0.001
Type 2 diabetes mellitus, *n* (%)	3 (3.3)	3 (2.5)	7 (8.3)	0.70	0.09
Nulliparous, *n* (%)	42 (45.2)	0 (0.0)	14 (17.7)		
Postmenopausal status, n (%)	93 (100)	92 (40.7)	25 (31.3)	<0.001	0.14
Age at menopause, years (s.d.)	34.9 (5.9)	47.6 (4.8)	46.9 (7.8)	<0.001	0.36
HRT ever used, *n* (%)	62 (72.1)	9 (11.8)	8 (30.8)	<0.001	0.03

Baseline characteristics were measured at time of the CT-scan. Baseline characteristics are summarized as means with standard deviation for normally distributed continuous data, as medians with interquartile ranges (IQR) for non-normally distributed continuous data and as numbers with percentages for categorical data.

^a^POI vs PE; ^b^PCOS vs PE.

PCOS, polycystic ovary syndrome; POI, premature ovarian insufficiency; PE, preeclampsia; BMI, body mass index; NE, North-European; HRT, hormone replacement therapy.

Missing values: waist circumference and central obesity, POI 28%, PE 7%, PCOS 11%. Dyslipidemia, POI 0%, PE 11%, PCOS 1%. Insulin resistance, POI 82%, PE 53%, PCOS 16%. Type 2 diabetes, POI 3%, PE 53%, PCOS 9%. Nulliparous, POI 0%, PE 0%, PCOS 14%. Postmenopausal status, POI 0%, PE 14%, PCOS 13%. Age at menopause, POI 13%, PE 87%, PCOS 86%. HRT ever used, POI 8%, PE 72%, PCOS 77%.

The PCOS group was older than the PE group (48.3 years (IQR 46.6–51.9) vs 46.0 years (IQR 43.4–49.0); *P* < 0.001). Fewer women with PCOS were postmenopausal compared to the PE group (31.3% vs 40.7%; *P* = 0.14).

### Trabecular bone density

**Figure 1:**
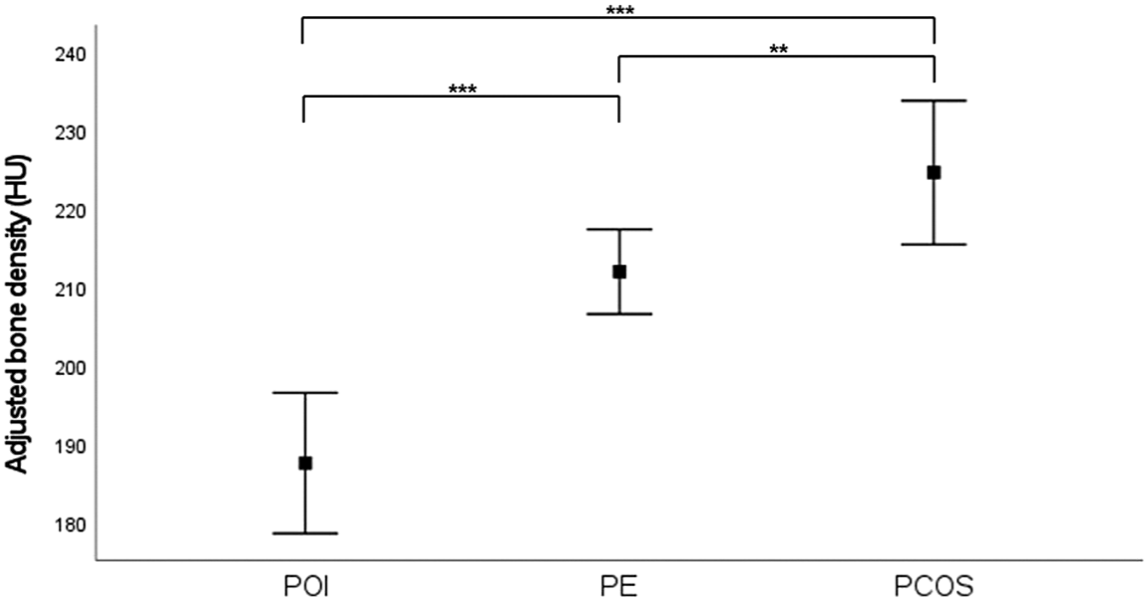
Adjusted trabecular bone density in women with reproductive disorders. Adjusted trabecular bone density expressed in HU values in women with POI, PE, and PCOS. Values are adjusted for age and BMI. POI has a mean TBD of 187.6 HU (95% CI 178.6–196.5), PE 211.9 (95% CI 206.6–217.3), and PCOS 224.6 (95% CI 215.4–233.7). ^*^P < 0.05, **P < 0.01, ***P < 0.001. PCOS, polycystic ovary syndrome; PE, preeclampsia; POI, premature ovarian insufficiency .

Women with POI have a lower TBD compared with women with PE (mean difference (MD) −34.4, 95% confidence interval (CI) −45.3 to −23.5). When correcting for age and BMI, women with POI still have a lower TBD compared to women with a history of PE (MD −24.4, 95% CI −35.0 to −13.8) (Fig. 1). Women with PCOS have a higher, but not significantly, TBD compared to women with a history of PE (MD 0.12, 95% CI −10.8 to 11.0). When correcting for age and BMI, this difference was significant (MD 12.6, 95% CI 1.8–23.4) (Fig. 1).

Sub-analysis in women with POI showed that TBD was not significantly different between women who ever used HRT and those who did not (*P* = 0.41) and that TBD was not significantly associated with age in this group (*P* = 0.71). Sub-analysis in women with PCOS showed that TBD was not significantly associated with central obesity, dyslipidemia, hypertension, insulin resistance, or type 2 diabetes mellitus. Age was significantly associated with TBD (*P* < 0.001).

## Discussion

In our study of middle-aged women with reproductive disorders, we found a positive trend of TBD in relation to the duration of estrogen exposure. Women with an reduced estrogen exposure (POI) had a lower TBD than women with an intermediate estrogen exposure (PE). Women with a prolonged estrogen exposure (PCOS) had the highest TBD.

The association between POI and decreased BD is well-established in literature. This association is explained by the loss of ovarian function early in life resulting in a decrease in estrogen levels (3, 4, 24, 25), for which HRT is recommended (6). As expected, in our study, women with POI had a lower TBD than women with a history of PE (intermediate group). Even though all women were diagnosed with POI in a university hospital and were therefore advised to use HRT, only 72.1% of them have ever used HRT. Despite HRT use, women with POI had lower TBD values. Unfortunately, we had no information about the duration of HRT use nor on the age of POI diagnosis, so we are not able to conclude whether the treatment was adequate. Another interesting finding was that TBD was not associated with age in the POI group as it was the case in the PCOS and PE group and has been described for the general population (14), this could also be explained by the reduced estrogen exposure.

Previous studies have shown contradictory results in the association between PCOS and BD (16, 17, 26, 27, 28). PCOS is, among others, associated with insulin resistance, central obesity, and hyperandrogenism (8, 9). Results from previous studies showed that insulin resistance, central obesity, dyslipidemia, and hypertension have a negative effect on BD (8, 9, 10, 11), and hyperandrogenism is described to increase BD (8, 28). In our study, women with PCOS had a higher TBD than women with PE, and insulin resistance and central obesity were not associated with TBD. In addition, a lower percentage of women with PCOS were postmenopausal even though they had a higher mean age. Therefore, we hypothesize that delayed menopause in PCOS has a protective effect on BD.

A strength of this study is that the TBD measurement is performed with an excellent intra- and interclass correlation. A limitation of our study is the missing data on covariates such as duration of hypogonadism and HRT use, type of HRT use, vitamin D status, estrogen, and androgen levels, history of bone fractures and family history of osteoporosis. Furthermore, in addition to the lack of a healthy control group in our study, there is also no population-based control group available because non-contrast CCT to measure BD is not the ‘gold standard’. In addition to a previously published study, we showed that CCTs can be used to assess both coronary artery calcium score for cardiovascular screening as well as TBD (29). This provides a unique opportunity in counseling women with reproductive disorders on long-term health issues.

## Declaration of interest

BCJM Fauser has received fees and/or grant support during the last 4 years from the following organizations (in alphabetic order): Bain Capital, Controversies in Obstetrics & Gynecology (COGI), Dutch Heart Foundation (Nederlandse Hartstichting), Elsevier, European Society of Human Reproduction and Embryology (ESHRE), Ferring, International Federation of Fertility Societies (IFFS), London Womens Clinic, Myovant, Netherlands Organisation for Health Research and Development (ZonMW), Pantharei Bioscience, Partners Group, PregLem/Gideon Richter, Shieldler, Reproductive Biomedicine Online (RBMO), UpToDate. JSE Laven has received fees and/or grant support during the last 4 years from the following organizations (in alphabetic order): Ansh Labs, Astellas, Ferring, Gedeon-Richter, Merck-Serono, National Institute of Health (NIH), Roche Diagnostics, and Titus Health Care.

## Funding

The CREw-IMAGO study is funded by the Dutch Heart Foundation (Grant 2013T083). The Dutch Heart Foundation had no role in the collection, analysis, and interpretation of data nor in the decision to submit the article for publication.

## Author contribution statement

JSEL, RPJB, BKV, AF, BCJMF, EB, and DB designed the study. CZ, AH, and KH performed the BD measurements and CZ and AH performed the descriptive analysis and drafted the manuscript. All authors were involved in interpreting the data, providing input to the manuscript, and approved the final manuscript. All authors read and approved the final manuscript.
